# Supplementary material for: Cardiomyocytes stimulate angiogenesis after ischemic injury in a ZEB2-dependent manner
Source: Nat Commun. 2021 Jan 4;12:84. doi: 10.1038/s41467-020-20361-3 (PMC7782784; doi:10.1038/s41467-020-20361-3)
Supplement: Supplementary file 1 — Supplementary Information [file 41467_2020_20361_MOESM1_ESM.pdf]

Supplementary information

Cardiomyocytes stimulate angiogenesis after ischemic injury in a ZEB2-dependent manner

Authors: Monika M. Gladka[1], Arwa Kohela[1], Bas Molenaar[1], Danielle Versteeg[1,2], Lieneke Kooijman[1], Jantine Monshouwer-Kloots[1], Veerle Kremer[3,4], Harmjan R. Vos[5], Manon M.H. Huibers[6], Jody J. Haigh[7], Danny Huylebroeck[8,9], Reinier A. Boon[3,10,11] Mauro Giacca[12], Eva van Rooij[1,2]*

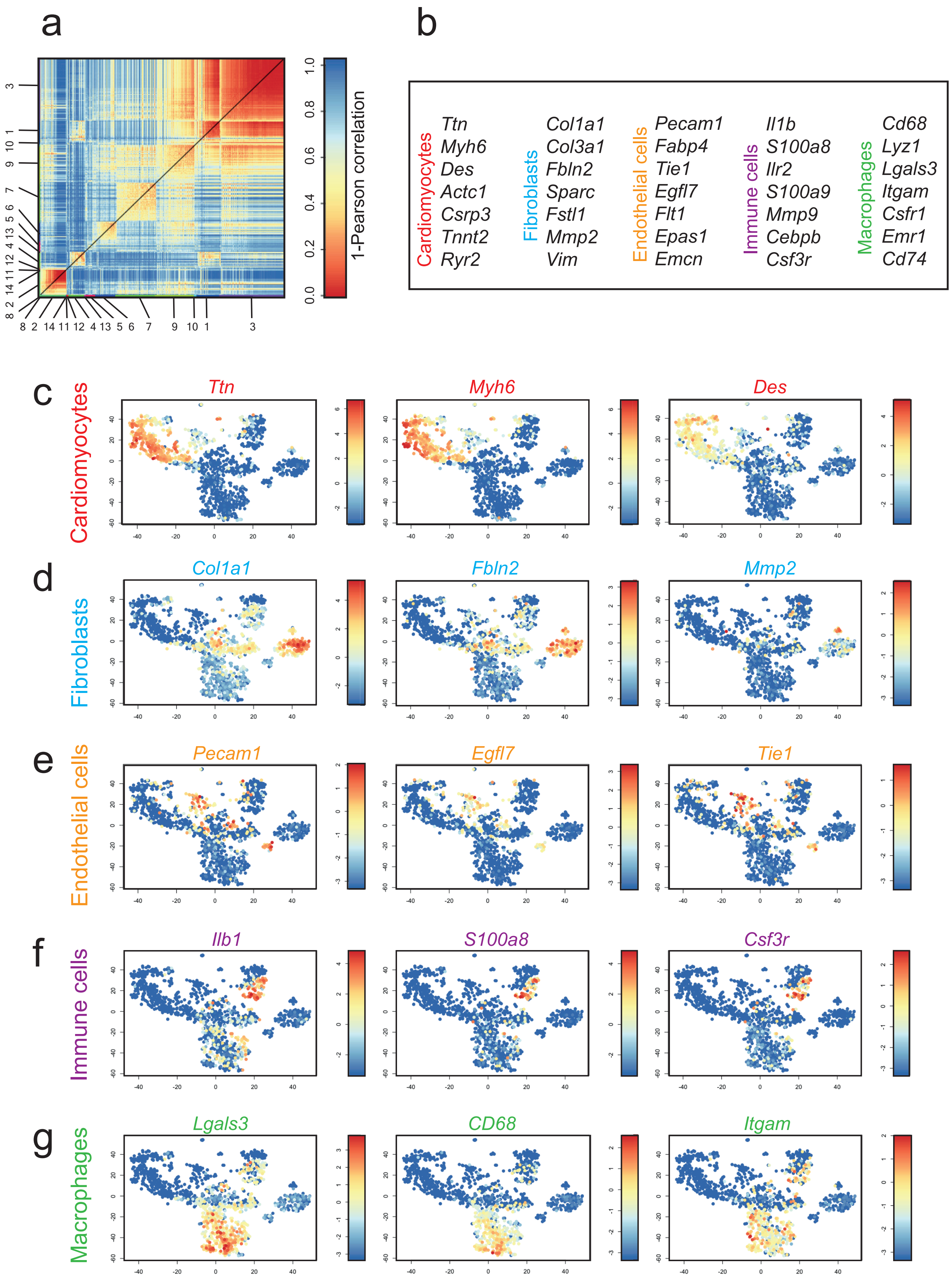

a
b
1-Pearson correlation
Cardiomyocytes
Ttn
Myh6
Des
Actc1
Csrp3
Tnnt2
Ryr2
Fibroblasts
Col1a1
Col3a1
Fbln2
Sparc
Fstl1
Mmp2
Vim
Endothelial cells
Pecam1
Fabp4
Tie1
Egfl7
Flt1
Epas1
Emcn
Immune cells
Il1b
S100a8
Ilr2
S100a9
Mmp9
Cebpb
Csf3r
Macrophages
Cd68
Lyz1
Lgals3
Itgam
Csfr1
Emr1
Cd74
c
Cardiomyocytes
Ttn
Myh6
Des
d
Fibroblasts
Col1a1
Fbln2
Mmp2
e
Endothelial cells
Pecam1
Egfl7
Tie1
f
Immune cells
Ilb1
S100a8
Csf3r
g
Macrophages
Lgals3
CD68
Itgam

Supplementary Figure 1. Clustering of cardiac cells based on gene expression differences. a Heatmap showing distances in cell-to-cell transcriptomes of cells obtained from sham and 3 dpMI hearts. Distances are measured by 1-Pearson's correlation coefficient.

K-medoids clustering identified 14 different cell clusters depicted on the x and y axes of the heatmap. **b** Table showing a list of known marker genes of main cardiac cell types used to identify the subpopulations of cells identified in **Fig. 1c. c-g** t-SNE maps indicating the expression of selected, well-established cellular markers in cell populations identified as **c** cardiomyocytes, **d** fibroblasts, **e** endothelial cells, **f** Immune cells, **g** macrophages. Data are shown as normalized transcript counts on a color-coded logarithmic scale. T-SNE indicates t-distributed stochastic neighbor embedding.

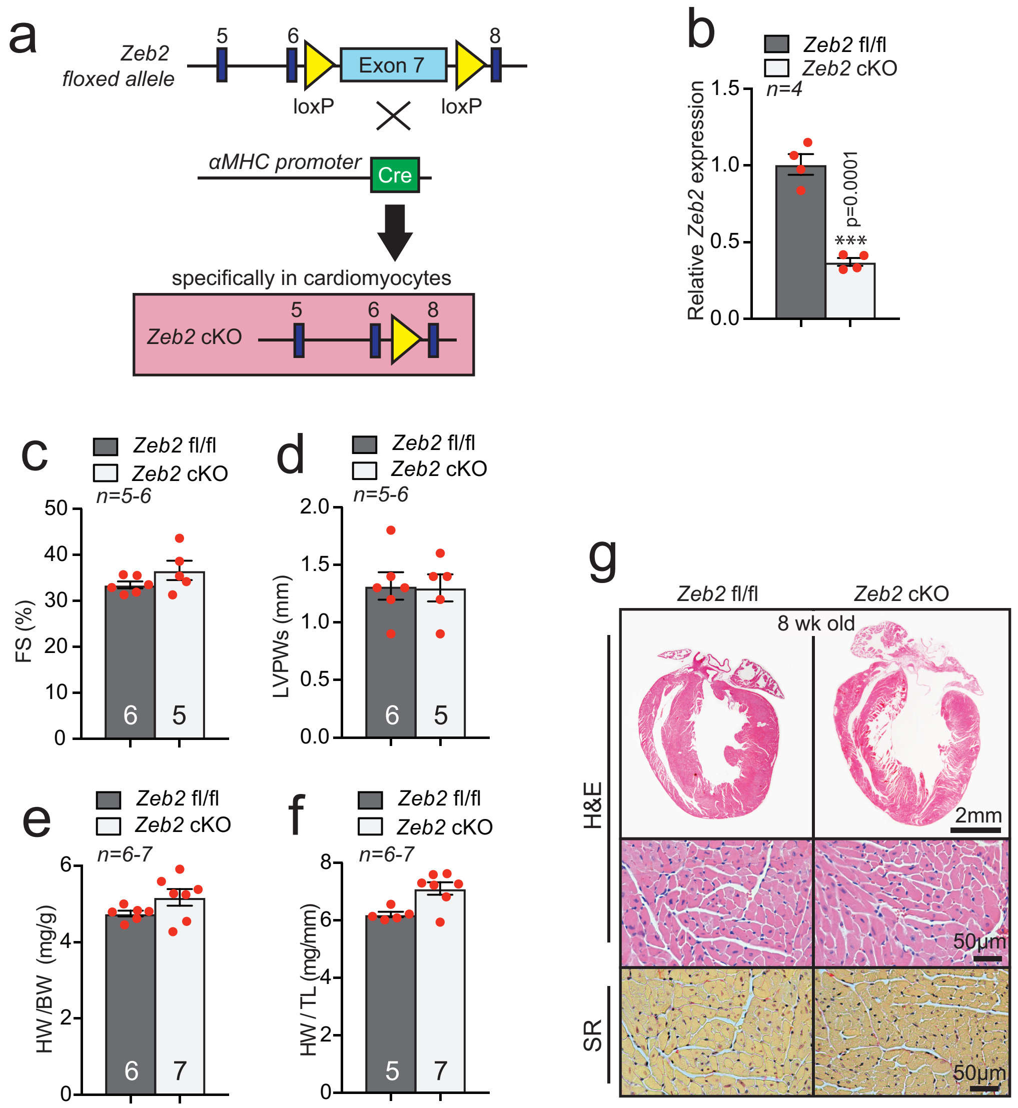

Supplementary Figure 2. Generation of a cardiomyocyte-specific Zeb2 cKO mouse model. **a** Schematic representation of generation of the Zeb2 cardiac KO (cKO). **b** mRNA expression level of Zeb2 in hearts from Zeb2 fl/fl and Zeb2 cKO mice. **c-d** Quantification of **c** fractional shortening (FS) and **d** left ventricular internal diameter in systole (LVIDs) from adult Zeb2 fl/fl and Zeb2 cKO mice. **e-f** Quantification of **e** HW/BW and **f** HW/TL ratio in Zeb2 fl/fl and Zeb2 ckO mice. **g** Representative images of four-chamber view (top panel), H&E stained sections (second panel) and SR stained sections (third panel) of hearts collected from adult Zeb2 fl/fl and Zeb2 cKO mice. H&E indicates Hematoxylin and Eosin, SR indicates Sirius Red, HW/BW indicates heart weight to body weight ratio, HW/TL indicates heart weight to tibia length ratio. Data are represented as mean ± SEM, ***p<0.0001 each dot indicates a biological replicate, n is indicated in figures. Comparison of two groups was performed with the unpaired, two-tailed Student's t-test between Zeb2 cKO vs Zeb2 fl/fl (**b, c, d, e, f**) Source data are provided as a Source Data file.

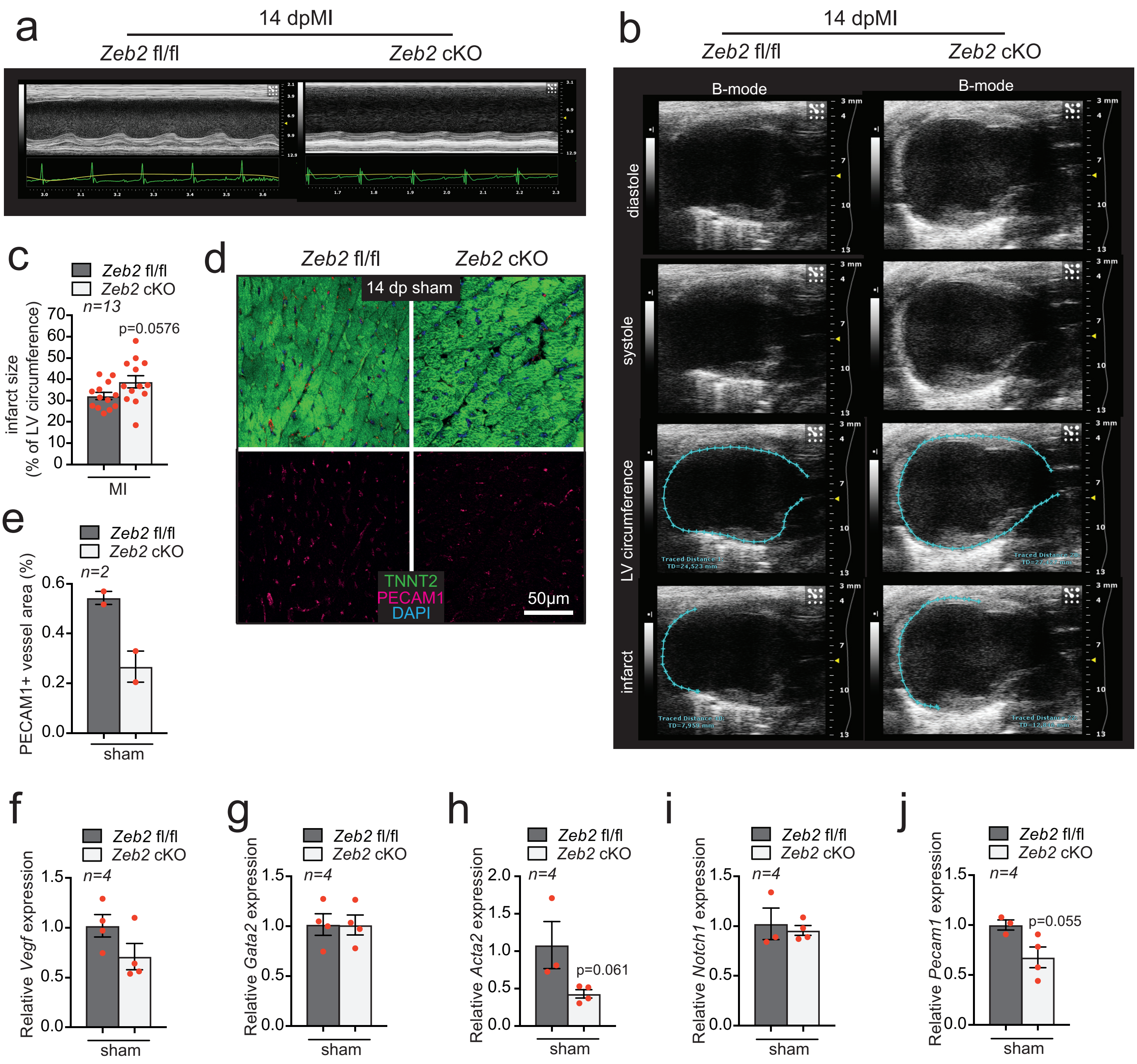

Supplementary Figure 3. Cardiomyocytes-specific ZEB2 deletion impairs cardiac function after injury. a Echocardiographic images of m-mode in hearts from Zeb2 fl/fl and Zeb2 cKO mice subjected to MI for 14 days. **b** Representative b-mode images of long access view in diastole and systole indicting infarcted region by measurements of percentage of LV circumference and **c** its quantification. **d** Immunofluorescence for PECAM1, TNNT2 and DAPI in Zeb2 fl/fl and Zeb2 cKO sham hearts **e** quantification of PECAM1 positive blood vessel area in histological sections from (**d**). Data in **d-e** from n=2. **f-j** mRNA expression levels of **f** Vegf and **g** Gata2 **h** Acta2 **i** Notch1 **j** Pecam1 in Zeb2 fl/fl and Zeb2 cKO sham mice. Data are represented as mean ± SEM, each dot indicates a biological replicate, n is indicated in figures. Comparison of two groups was performed with the unpaired, two-tailed Student's t-test between Zeb2 cKO vs Zeb2 fl/fl post-MI (**c**) or post-sham (**f, g, h, i, j**). Source data are provided as a Source Data file.

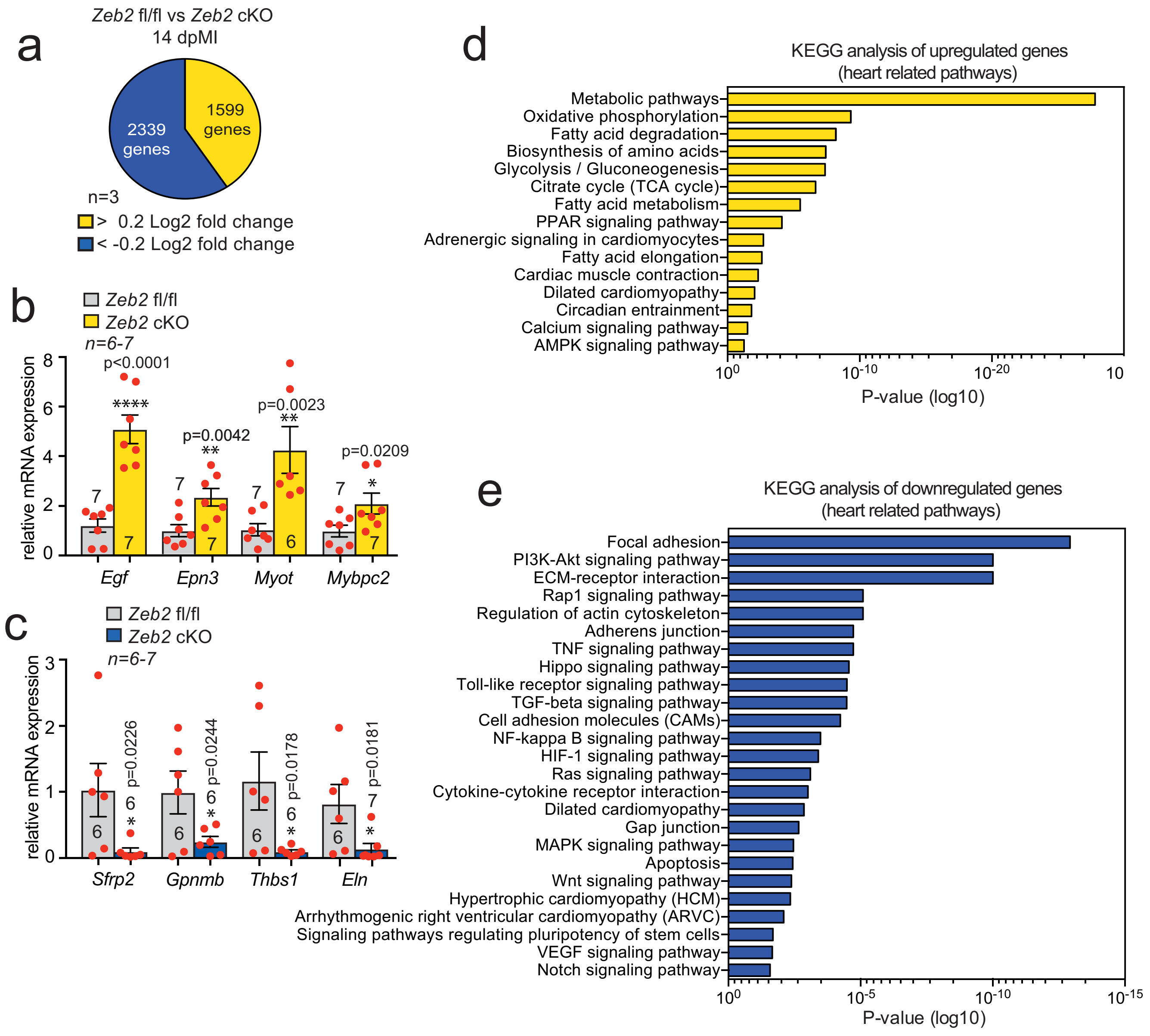

Supplementary Figure 4. RNA-seq to identify the role of ZEB2 in the injured heart. a Representation of the RNA-seq dataset as pie diagram showing down- and upregulated genes in Zeb2 fl/fl and Zeb2 cKO heart tissue 14 dpMI. **b-c** mRNA expression levels of **b** upregulated and **c** downregulated genes. **d-e** KEGG pathway analysis on genes significantly **d** upregulated and **e** downregulated in Zeb2 cKO hearts when compared to Zeb2 fl/fl hearts post injury. Data are represented as mean ± SEM, *p<0.05, **p<0.01, ****p<0.0001, each dot indicates a biological replicate, n is indicated in figures. Comparison of two groups was performed with the unpaired, two-tailed Student's t-test between Zeb2 cKO vs Zeb2 fl/fl post-MI (**b, c**). Source data are provided as a Source Data file.

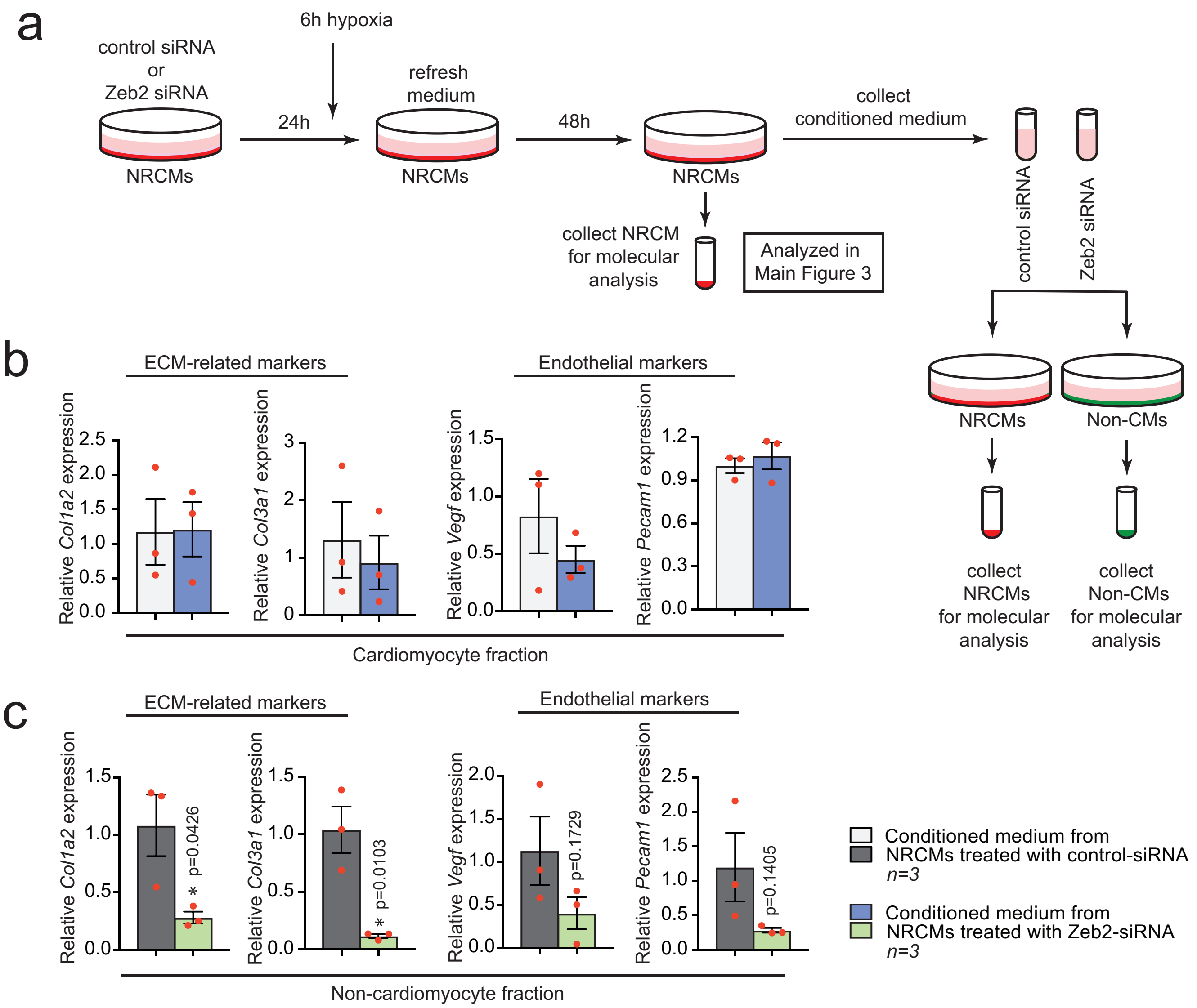

Supplementary Figure 5. Cellular communication is ZEB2 dependent. a Experimental setup to produce and collect conditioned medium from NRCMs treated with ZEB2 silencing RNA. **b-c** mRNA expression levels of fibroblast and endothelial markers in **b** NRCMs **c** non-cardiomyocytes treated with conditioned media collected in (**a**). Data are represented as mean ± SEM, *p<0.05, each dot indicates a biological replicate, n is indicated in figures. Comparison of two groups was performed with the unpaired, two-tailed Student's t-test (**b, c**). Source data are provided as a Source Data file.

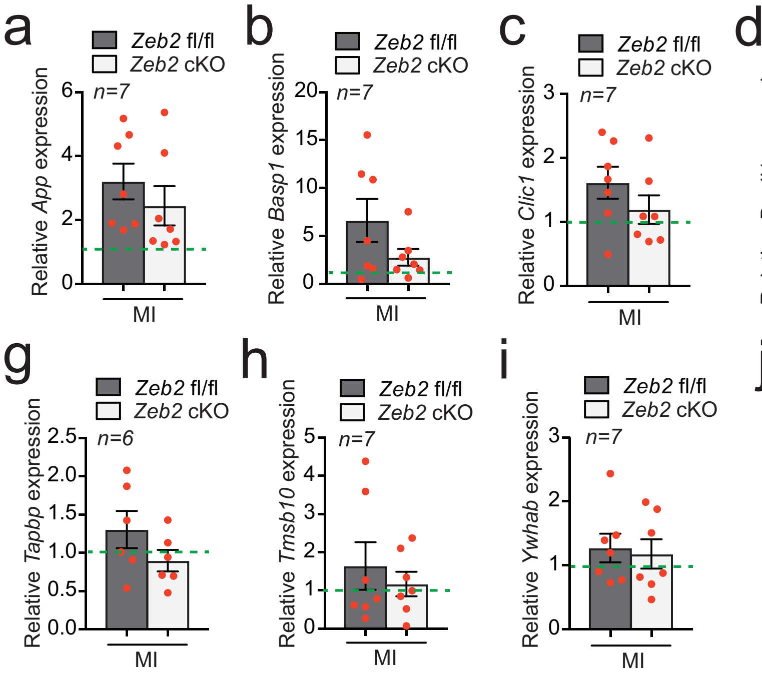

j

10 kb upstream and 10kb downstream of TSS

Mouse		Human	
gene name	ZEB2 motifs	gene name	ZEB2 motifs
Tmsb4x	8	*TMSB4X*	11
Ptma	40	*PTMA*	38

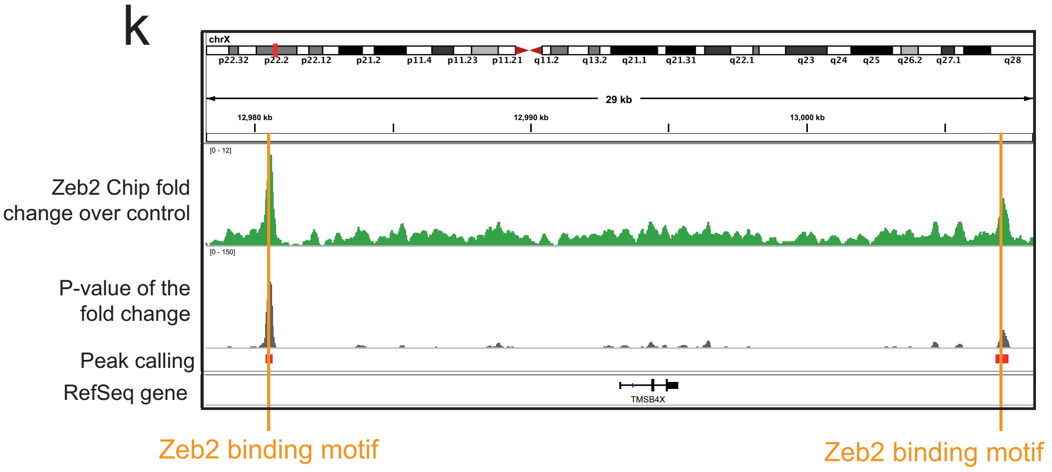

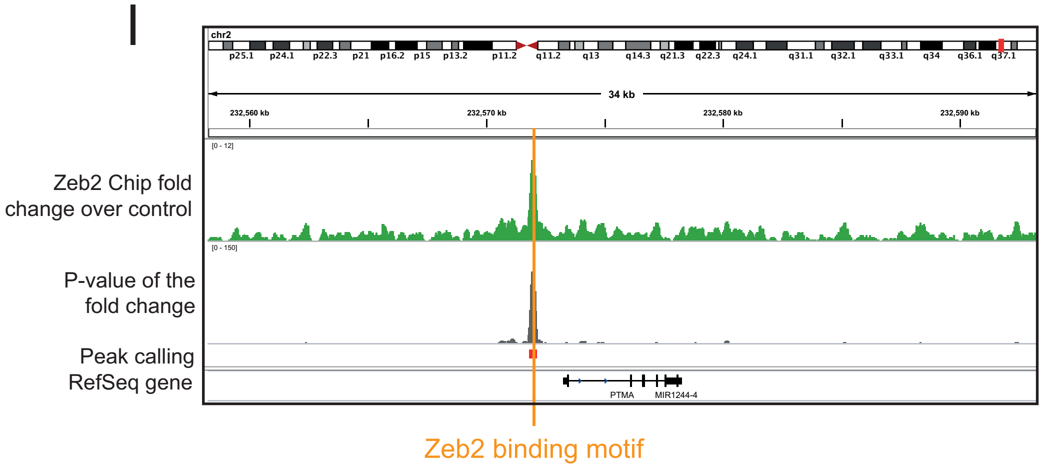

Supplementary Figure 6. Validation of factors identified by mass spectrometry in vivo. a-i mRNA expression levels of **a** App, **b** Basp1, **c** Clic1, **d** Dynll1, **e** Rack1, **f** Spcs2, **g** Tapbp, **h** Tmsb10, **i** Ywhab in hearts from Zeb2 fl/fl and Zeb2 cKO mice. **j** ZEB2 predicted binding sites in 10kb upstream and 10kb downstream region of the transcription starting site (TSS) of Tmsb4 and Ptma in mouse and human. **k-l** ZEB2 protein directly binds to **k** TMSB4 and **l** PTMA promoters. Green dashed line indicates sham Zeb2 fl/fl control. Orange line denotes ZEB2 binding motifs. Data are represented as mean ± SEM, $*p<0.05$, $***p<0.001$, each dot indicates a biological replicate, n is indicated in figures. Comparison of two groups was performed with the unpaired, two-tailed Student's t-test between Zeb2 cKO vs Zeb2 fl/fl groups post-MI (**a, b, c, d, e, f, g, h, i**). Source data are provided as a Source Data file.

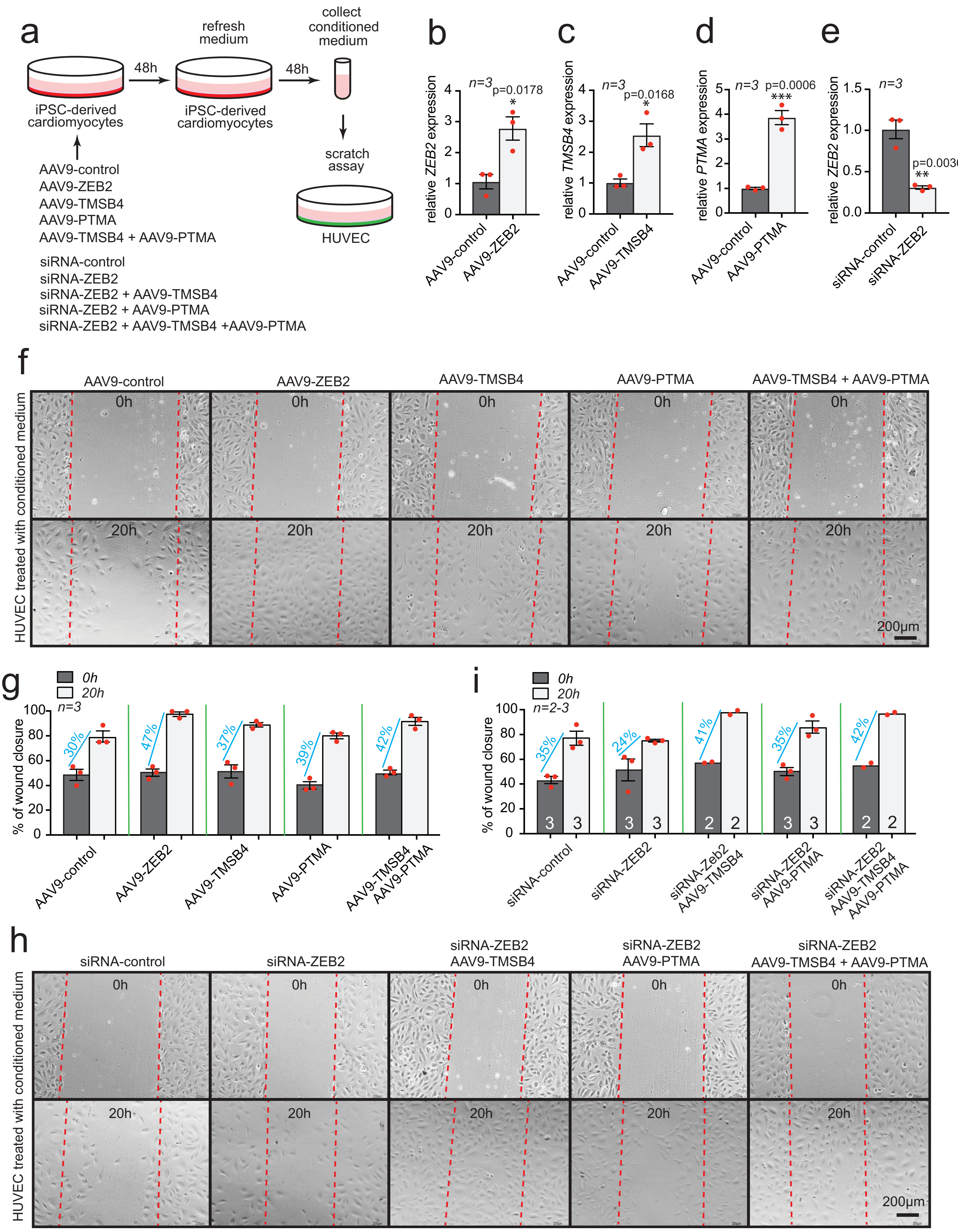
a
refresh medium
collect conditioned medium
48h
48h
iPSC-derived cardiomyocytes
iPSC-derived cardiomyocytes
scratch assay
HUVEC
AAV9-control
AAV9-ZEB2
AAV9-TMSB4
AAV9-PTMA
AAV9-TMSB4 + AAV9-PTMA
siRNA-control
siRNA-ZEB2
siRNA-ZEB2 + AAV9-TMSB4
siRNA-ZEB2 + AAV9-PTMA
siRNA-ZEB2 + AAV9-TMSB4 +AAV9-PTMA
b
relative ZEB2 expression
n=3
p=0.0178
c
relative TMSB4 expression
n=3
p=0.0168
d
relative PTMA expression
n=3
p=0.0006
e
relative ZEB2 expression
n=3
p=0.0036
f
HUVEC treated with conditioned medium
0h
20h
200µm
g
0h
20h
n=3
% of wound closure
30%
47%
37%
39%
42%
AAV9-TMSB4 AAV9-PTMA
i
n=2-3
35%
24%
41%
35%
42%
siRNA-Zeb2 AAV9-TMSB4
siRNA-ZEB2 AAV9-PTMA
siRNA-ZEB2 AAV9-TMSB4 AAV9-PTMA
h
siRNA-ZEB2 AAV9-TMSB4 + AAV9-PTMA

Supplementary Figure 7. Expression of ZEB2, TMSB4 and PTMA in cardiomyocytes drives endothelial cell migration. a Conditioned medium experimental setup. **b-e** mRNA expression levels of (**b), e** ZEB2, **c** TMSB4 **d** PTMA in iPSC-derived cardiomyocytes treated with AAV9-control, AAV9-ZEB2, AAV9-TMSB4, AAV9-PTMA or siRNA-control and siRNA ZEB2, respectively **f** Representative images of scratch assay in HUVECs treated with indicated conditioned medium at 0 and 20 hours after treatment. **g** Quantification of wound closure from (**f**). **h** Representative images of scratch assay in HUVECs treated with the indicated conditioned media at 0 and 20 hours after treatment. **i** Quantification of wound closure from (**h**). Data in **f-h** is from one experiment with 2-3 biological replicates. Data are represented as mean ± SEM, *$p<0.05$, ***$p<0.001$, each dot indicates a biological replicate, n is indicated in figures. Comparison of two groups was performed with the unpaired, two-tailed Student's t-test (**b, c, d, e**). Source data are provided as a Source Data file.

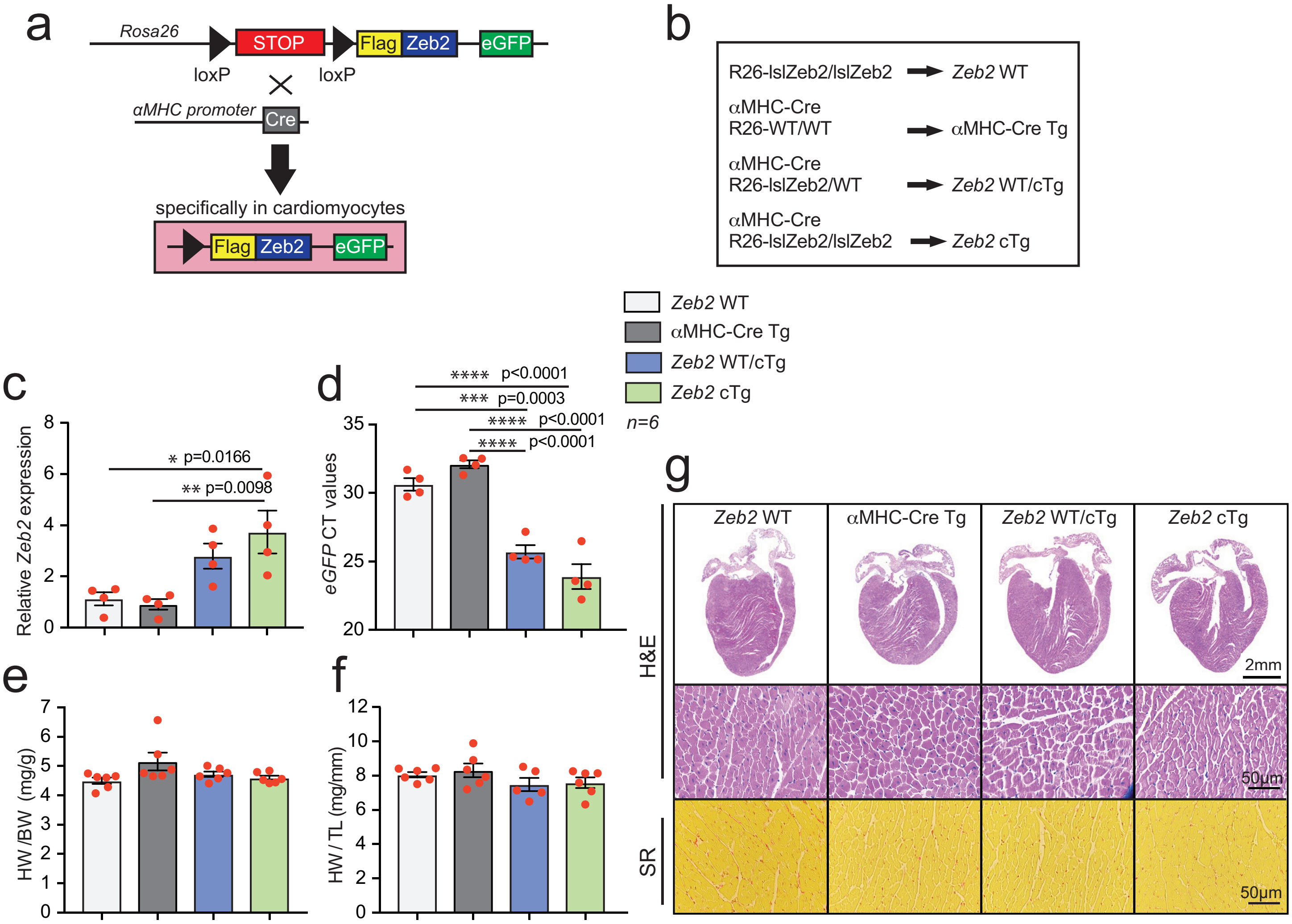

Supplementary Figure 8. Generation of a cardiomyocyte-specific Zeb2 cTg mouse model. a Schematic representation of generation of the ZEB2 cardiac overexpression model (Zeb2 cTg). **b** Detailed description of the used genotypes and their simplified names. **c-d** mRNA expression levels of **c** Zeb2 and **d** eGFP (CT values) in hearts from Zeb2 WT and Zeb2 cTg mice. **e-f** Quantification of **e** HW/BW and **f** HW/TL ratio in Zeb2 WT and Zeb2 cTg mice. **g** Representative images of four-chamber view (top panel), H&E stained sections (second panel) and SR stained sections (third panel) of hearts collected from adult Zeb2 WT and Zeb2 cTg mice. H&E indicates Hematoxylin and Eosin, SR indicates Sirius Red, HW/BW indicates heart weight to body weight ratio, HW/TL indicates heart weight to tibia length ratio. Data are represented as mean ± SEM, *p<0.05, **p<0.01, ***p<0.001, ****p<0.0001, each dot indicates a biological replicate, n is indicated in figures. Data were analysed by ordinary one-way ANOVA with Tukey multiple comparison test (**c, d, e, f**) Source data are provided as a Source Data file.

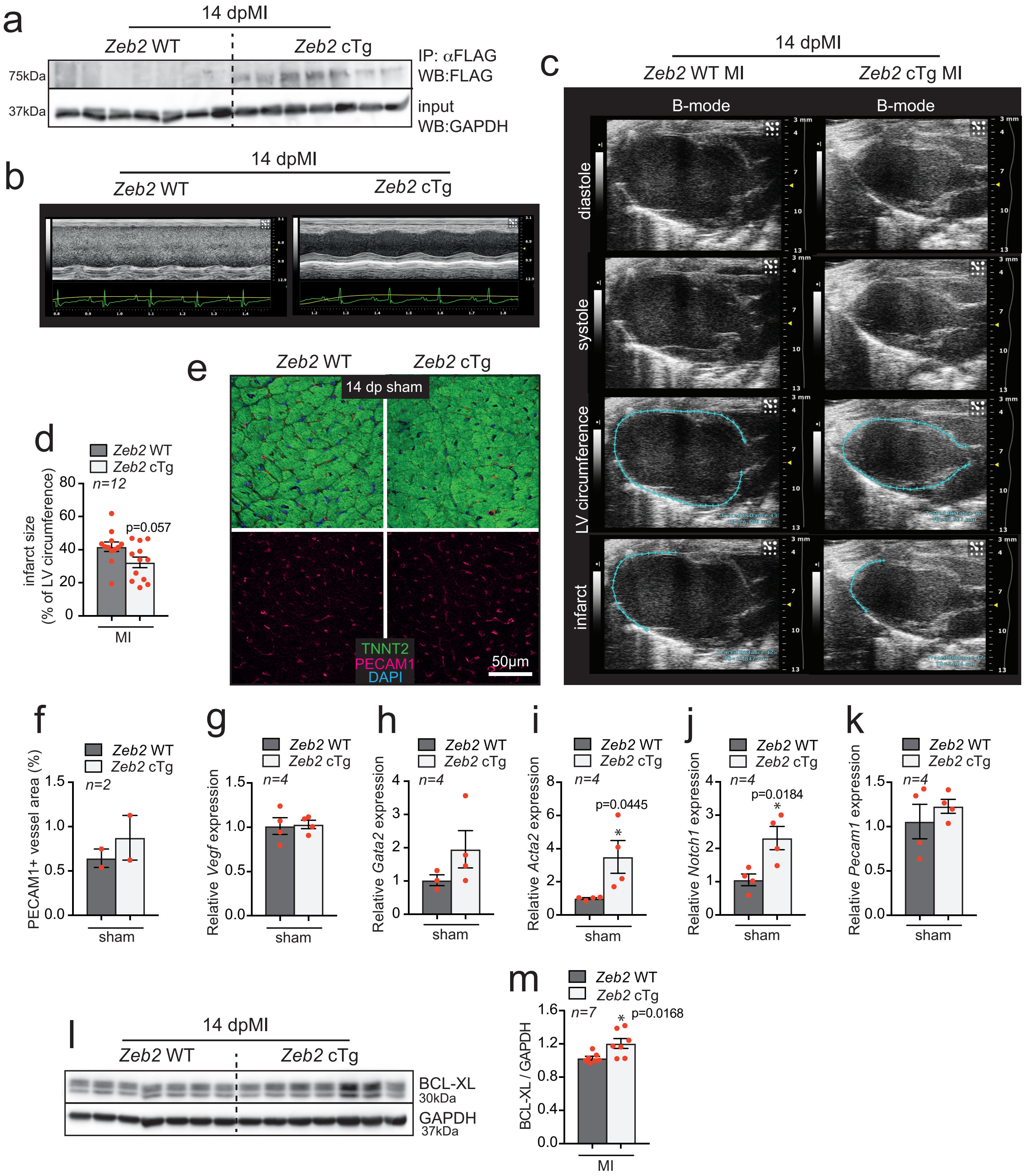
a
14 dpMI
Zeb2 WT
Zeb2 cTg
75kDa
37kDa
IP: αFLAG
WB:FLAG
input
WB:GAPDH
b
14 dpMI
Zeb2 WT
Zeb2 cTg
c
14 dpMI
Zeb2 WT MI
Zeb2 cTg MI
B-mode
B-mode
diastole
systole
LV circumference
infarct
d
Zeb2 WT
Zeb2 cTg
n=12
p=0.057
infarct size
(% of LV circumference)
MI
e
Zeb2 WT
Zeb2 cTg
14 dp sham
TNNT2
PECAM1
DAPI
50µm
f
Zeb2 WT
Zeb2 cTg
n=2
PECAM1+ vessel area (%)
sham
g
Zeb2 WT
Zeb2 cTg
n=4
Relative Vegf expression
sham
h
Zeb2 WT
Zeb2 cTg
n=4
Relative Gata2 expression
sham
i
Zeb2 WT
Zeb2 cTg
n=4
p=0.0445
Relative Acta2 expression
sham
j
Zeb2 WT
Zeb2 cTg
n=4
p=0.0184
Relative Notch1 expression
sham
k
Zeb2 WT
Zeb2 cTg
n=4
Relative Pecam1 expression
sham
l
14 dpMI
Zeb2 WT
Zeb2 cTg
BCL-XL
30kDa
GAPDH
37kDa
m
Zeb2 WT
Zeb2 cTg
n=7
p=0.0168
BCL-XL / GAPDH
MI

Supplementary Figure 9. Cardiomyocyte-specific ZEB2 overexpression protects cardiac function after MI. a FLAG immunoprecipitation in heart tissue from Zeb2 WT and Zeb2 cTg 14 dpMI. **b** Echocardiographic images of m-mode in hearts from Zeb2 WT and Zeb2 cTg mice subjected to MI for 14 days. **c** Representative b-mode images of long access view in diastole and systole indicting infarcted region by measurements of percentage of LV circumference and **d** its quantification. **e** Immunofluorescence for PECAM1, TNNT2 and DAPI in Zeb2 WT and Zeb2 cTg sham hearts **f** quantification of PECAM1 positive blood vessel area in histological sections from (**e**). Data in **e-f** from n=2. **g-k** mRNA expression levels of **g** Vegf and **h** Gata2 **i** Acta2 **j** Notch1 **k** Pecam1 in Zeb2 WT and Zeb2 cTg sham mice. **l** Representative WB for BCL-XL in tissue from Zeb2 WT and Zeb2 cTg 14 dpMI and **m** its quantification. Data are represented as mean ± SEM, *p<0.05, each dot indicates a biological replicate, n is indicated in figures. Comparison of two groups was performed with the unpaired, two-tailed Student's t-test between Zeb2cTg vs Zeb2 WT post-MI (**d, m**) or post-sham (**f, g, h, i, j, k**). Source data are provided as a Source Data file.

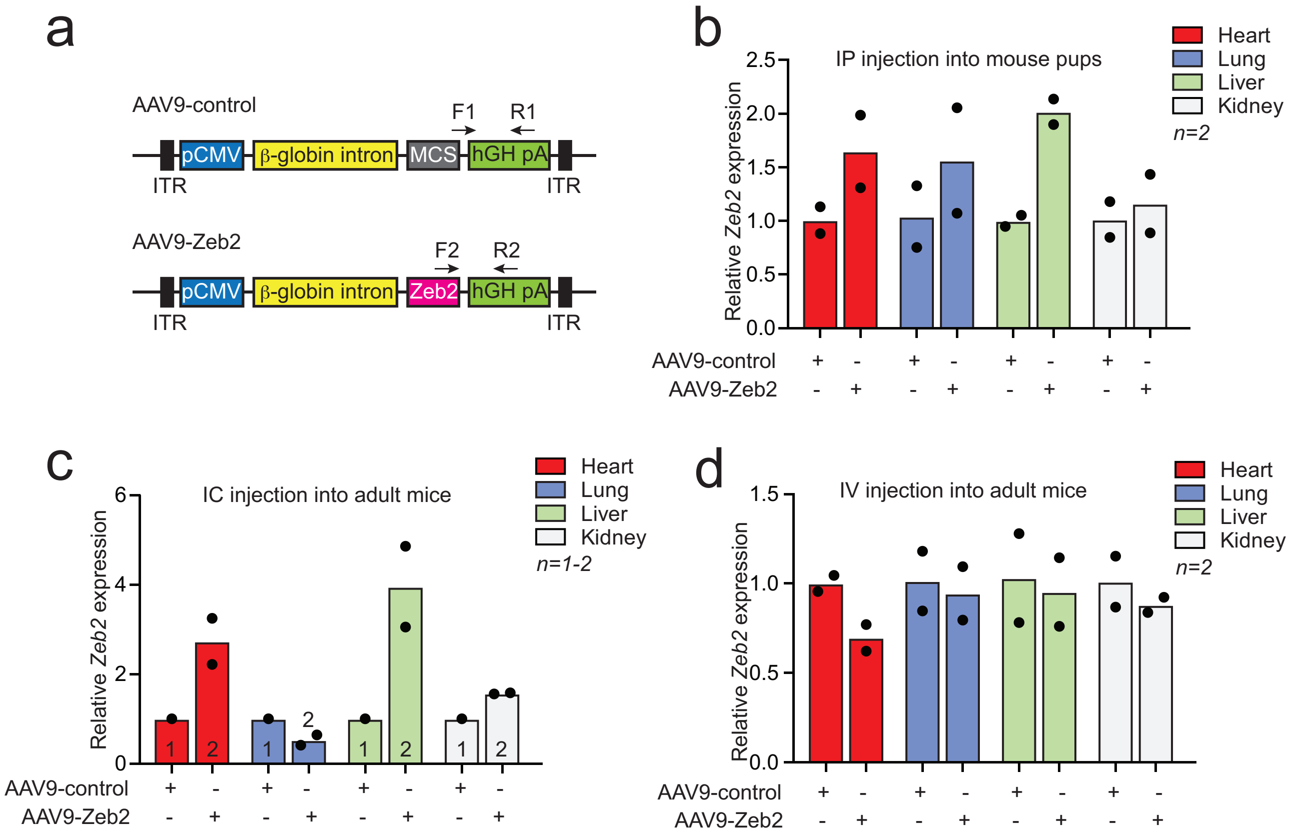

Supplementary Figure 10. Efficiency of AAV9-Zeb2 delivery in vivo. a Schematic representation of constructs used to generate AAV9 viruses. **b-d** mRNA expression level of Zeb2 in the indicated organs of **b** intraperitoneal (IP), **c** intracardiac (IC) and **d** intravenous (IV) AAV9-Zeb2-injected mice. Data are represented as mean ± SEM, each dot indicates a biological replicate, n is indicated in figures. Source data are provided as a Source Data file.

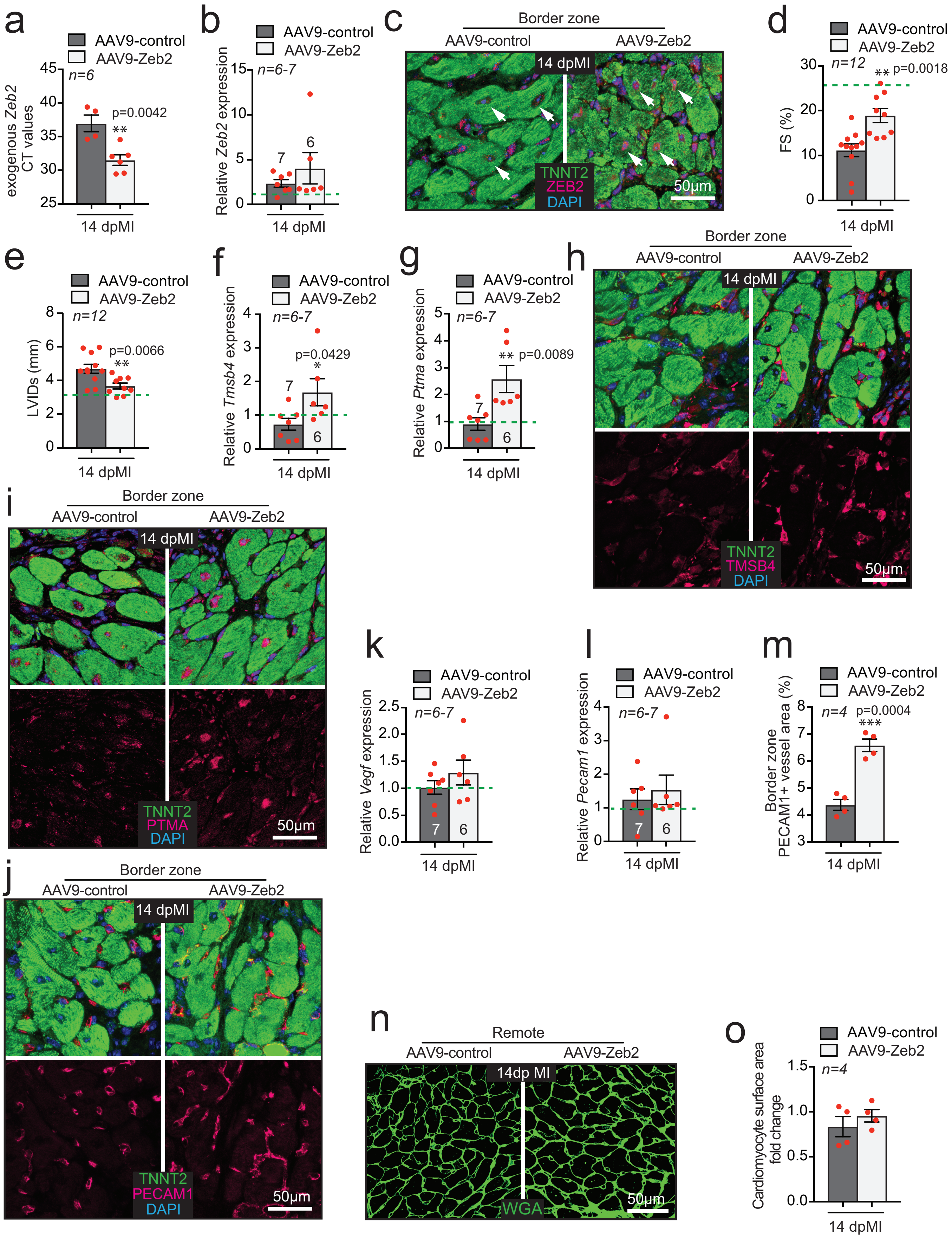
a
AAV9-control
AAV9-Zeb2
exogenous Zeb2 CT values
n=6
p=0.0042
**
14 dpMI
b
Relative Zeb2 expression
n=6-7
14 dpMI
c
Border zone
AAV9-control
AAV9-Zeb2
14 dpMI
TNNT2
ZEB2
DAPI
50µm
d
FS (%)
n=12
** p=0.0018
14 dpMI
e
LVIDs (mm)
n=12
p=0.0066
**
14 dpMI
f
Relative Tmsb4 expression
n=6-7
p=0.0429
*
14 dpMI
g
Relative Ptma expression
n=6-7
** p=0.0089
14 dpMI
h
Border zone
14 dpMI
TNNT2
TMSB4
DAPI
50µm
i
Border zone
14 dpMI
TNNT2
PTMA
DAPI
50µm
j
Border zone
14 dpMI
TNNT2
PECAM1
DAPI
50µm
k
Relative Vegf expression
n=6-7
14 dpMI
l
Relative Pecam1 expression
n=6-7
14 dpMI
m
Border zone PECAM1+ vessel area (%)
n=4
p=0.0004
***
14 dpMI
n
Remote
AAV9-control
AAV9-Zeb2
14dp MI
WGA
50µm
o
Cardiomyocyte surface area fold change
n=4
14 dpMI

Supplementary Figure 11. AAV9-mediated ZEB2 delivery is cardioprotective. a-b Expression levels of **a** exogenous Zeb2 and **b** Zeb2 mRNA in AAV9-control and AAV9-Zeb2 treated mice 14 days post-surgery. **c** Immunohistochemistry for ZEB2, TNNT2 and DAPI nuclei of histological sections of hearts from AAV9-control and AAV9-Zeb2 treated mice 14 dpMI (border zone). **d-e** Quantification of **d** fractional shortening (FS) and **e** left ventricular internal diameter in systole (LVIDs) from AAV9-control and AAV9-Zeb2 treated mice 14 dpMI. **f-g** mRNA expression levels of **f** Tmsb4 and **g** Ptma in hearts from AAV9-control and AAV9-Zeb2 treated mice 14 days post-MI. **h-i** Immunofluorescence for **h** TMSB4 or **i** PTMA, ACTN2 and DAPI nuclei of histological sections of hearts from AAV9-control and AAV9-Zeb2 treated mice 14 dpMI. **j** Immunofluorescence for PECAM1, TNNT2 and DAPI nuclei of histological sections of hearts from AAV9-control and AAV9-Zeb2 treated mice 14 dpMI. **k-l** mRNA expression level of **k** Vegf and **l** Pecam1 in hearts from AAV9-control and AAV9-Zeb2 treated mice 14 dpMI. **m** Quantification of PECAM1 positive blood vessel area in histological sections of hearts from AAV9-control and AAV9-Zeb2 treated mice 14 dpMI. **n** WGA staining to measure cardiomyocyte surface area and **o** its quantification. Green dashed line indicates sham AAV9-control. White arrows show ZEB2 positive cardiomyocytes. Data are represented as mean ± SEM, *$p<0.05$, **$p<0.01$, ***$p<0.001$, each dot indicates a biological replicate, n is indicated in figures. Comparison of two groups was performed with the unpaired, two-tailed Student's t-test between AAV9-Zeb2 vs AAV9-control post-MI (**a, b, d, e, f, g, k, l, m, o**). Source data are provided as a Source Data file.

Supplementary Table 1. Morphometric and echocardiographic characteristic of Zeb2 fl/fl and Zeb2 cKO mice.

	Zeb2 fl/fl	*Zeb2* cKO
**n**	6	6
**BW (g)**	20,8 ± 1,99	21,7 ± 2,28
**HW (g)**	0,10 ± 0,01	0,11 ± 0,01
**TL (mm)**	15,3 ± 0,53	15,7 ± 0,55
**IVSd (mm)**	0,86 ± 0,13	0,94 ± 0,24
**IVSs (mm)**	1,33 ± 0,12	1,42 ± 0,2
**LVIDd (mm)**	3,83 ± 0,13	3,94 ± 0,23
**LVIDs (mm)**	2,56 ± 0,16	2,6 ± 0,25
**LVPWd (mm)**	0,93 ± 0,17	0,9 ± 0,25
**LVPWs (mm)**	1,31 ± 0,29	1,3 ± 0,26
**EF (%)**	62,8 ± 2,6	66,5 ± 5,4
**FS (%)**	33,4 ± 1,8	36,6 ± 4,6
**Heart rate**	435 ± 24	436 ± 25

BW, body weight; HW, heart weight; TL, tibia length; IVSd, interventricular septal thickness at end-diastole; IVSs, interventricular septal thickness at end-systole; LVIDd, left ventricular internal dimension at end-diastole; LVIDs, left ventricular internal dimension at end-systole; LVPWd, left ventricular posterior wall thickness at end-diastole; LVPWs, left ventricular posterior wall thickness at end-systole; EF, ejection fraction; FS, fractional shortening; Data are expressed as means ± STDEV. Comparison of two groups was performed with the unpaired, two-tailed Student’s t-test between Zeb2 cKO vs Zeb2 fl/fl.

Supplementary Table 2. Morphometric and echocardiographic characteristic of Zeb2 fl/fl and Zeb2 cKO mice subjected to sham or MI for 14 days.

	Zeb2 fl/fl	*Zeb2* cKO
**Surgery**	sham	sham
**n**	6	7
**BW (g)**	26,1 ± 2,47	28 ± 1,22
**HW (g)**	0,13 ± 001	0,13 ± 0,01
**TL (mm)**	16,3 ± 0,63	16,7 ± 0,35
**IVSd (mm)**	1,71 ± 0,55	1,33 ± 0,35
**IVSs (mm)**	2,17 ± 0,49	1,79 ± 0,42
**LVIDd (mm)**	3,78 ± 0,52	4,08 ± 0,54
**LVIDs (mm)**	2,58 ± 0,62	2,9 ± 0,71
**LVPWd (mm)**	1,22 ± 0,28	1,1 ± 0,13
**LVPWs (mm)**	1,74 ± 0,35	1,39 ± 0,29
**EF (%)**	61,6 ± 10,3	59,9 ± 11,6
**FS (%)**	33,1 ± 7,3	32,1 ± 7,9
**Heart rate**	411 ± 44	407 ± 29
	Zeb2 fl/fl	*Zeb2* cKO
**Surgery**	MI	MI
**n**	10	11
**BW (g)**	28,9 ± 1,65	28,4 ± 2,84
**HW (g)**	0,17 ± 0,02	0,19 ± 0,04
**TL (mm)**	16,7 ± 0,53	16,4 ± 0,54
**IVSd (mm)**	1,26 ± 0,44	1,05 ± 0,27
**IVSs (mm)**	1,60 ± 0,63	1,23 ± 0,35
**LVIDd (mm)**	5,10 ± 0,89 $$ p=0.0094	5,41 ± 0,65
**LVIDs (mm)**	4,05 ± 1,09 $ p=0.0227	4,62 ± 0,84
**LVPWd (mm)**	1,31 ± 0,44	1,15 ± 0,47
**LVPWs (mm)**	1,54 ± 0,39	1,37 ± 0,57
**EF (%)**	44,7 ± 13,6	32,7 ± 14,5 # p=0.0450
**FS (%)**	23,5 ± 8,4 $ p=0.0382	16,1 ± 7,6 # p=0.0238
**Heart rate**	415 ± 42	424 ± 28

sham, sham-operated control group; MI, myocardial infarction; BW, body weight; HW, heart weight; TL, tibia length; IVSd, interventricular septal thickness at end-diastole; IVSs, interventricular septal thickness at end-systole; LVIDd, left ventricular internal dimension at end-diastole; LVIDs, left ventricular internal dimension at end-systole; LVPWd, left ventricular posterior wall thickness at end-diastole; LVPWs, left ventricular posterior wall thickness at end-systole; EF, ejection fraction; FS, fractional shortening; Data are expressed as means ± STDEV. Comparison of two groups was performed with the unpaired, two-tailed Student's t-test between Zeb2 fl/fl post-MI vs Zeb2 fl/fl post-sham (indicated by dollar sign, $p<0.05, $$p<0.01) and Zeb2 cKO post-MI vs Zeb2 fl/fl post-MI (indicated by pound sign, #p<0.05).

Supplementary Table 3. Morphometric and echocardiographic characteristic of Zeb2 WT, αMHC-Cre Tg, Zeb2 WT/cTg and Zeb2 cTg mice.

	Zeb2 WT	aMHC-Cre Tg	*Zeb2* WT/cTg	*Zeb2* cTg
**n**	6	6	6	6
**BW (g)**	31,4 ± 1,46	28,48 ± 1,36	28,45 ± 2,5	28,71 ± 2,47
**HW (g)**	0,14 ± 0,01	0,15 ± 0,02	0,13 ± 0,01	0,13 ± 0,01
**TL (mm)**	17,51 ± 0,37	17,61 ± 0,17	17,48 ± 0,44	17,41 ± 0,29
**IVSd (mm)**	0,94 ± 0,1	0,98 ± 0,15	0,91 ± 0,05	0,84 ± 0,03
**IVSs (mm)**	1,23 ± 0,11	1,31 ± 0,18	1,25 ± 0,11	1,72 ± 0,11
**LVIDd (mm)**	3,98 ± 0,27	4,06 ± 0,12	4,14 ± 0,20	4,10 ± 0,36
**LVIDs (mm)**	2,79 ± 0,41	3,01 ± 0,38	3,03 ± 0,25	2,91 ± 0,39
**LVPWd (mm)**	0,96 ± 0,08	1,15 ± 0,3	0,93 ± 0,22	1,01 ± 0,17
**LVPWs (mm)**	1,24 ± 0,12	1,48 ± 0,42	1,21 ± 0,19	1,29 ± 0,21
**EF (%)**	57,52 ± 8,88	50,91 ± 12,27	52,61 ± 8,29	56,30 ± 7,09
**FS (%)**	30,10 ± 6,07	26,08 ± 7,88	26,92 ± 5,35	29,21 ± 4,47
**Heart rate**	429 ± 32	416 ± 29	376 ± 19	368 ± 50

BW, body weight; HW, heart weight; TL, tibia length; IVSd, interventricular septal thickness at end-diastole; IVSs, interventricular septal thickness at end-systole; LVIDd, left ventricular internal dimension at end-diastole; LVIDs, left ventricular internal dimension at end-systole; LVPWd, left ventricular posterior wall thickness at end-diastole; LVPWs, left ventricular posterior wall thickness at end-systole; EF, ejection fraction; FS, fractional shortening; Data are expressed as means ± STDEV. Data were analyzed by ordinary one-way ANOVA with Sidak’s multiple comparison test.

Supplementary Table 4. Morphometric and echocardiographic characteristic of Zeb2 WT and Zeb2 cTg mice subjected to MI for 14 days.

	Zeb2 WT	*Zeb2* cTg
**Surgery**	sham	sham
**n**	6	6
**BW (g)**	30,2 ± 2,68	30,34 ± 2,89
**HW (g)**	0,14 ± 0,01	0,15 ± 0,03
**TL (mm)**	17,47 ± 0,38	17,16 ± 0,42
**IVSd (mm)**	0,77 ± 0,13	0,81 ± 0,09
**IVSs (mm)**	1,13 ± 0,14	1,22 ± 0,14
**LVIDd (mm)**	3,79 ± 0,15	3,99 ± 0,35
**LVIDs (mm)**	2,78 ± 0,17	2,77 ± 0,46
**LVPWd (mm)**	0,94 ± 0,18	0,79 ± 0,08
**LVPWs (mm)**	1,17 ± 0,2	1,11 ± 0,17
**EF (%)**	52,72 ± 6,03	58,33 ± 11,82
**FS (%)**	26,68 ± 3,85	30,88 ± 8,03
**Heart rate**	403 ± 31	342 ± 73
	Zeb2 WT	*Zeb2* cTg
**Surgery**	MI	MI
**n**	11	11
**BW (g)**	29,89 ± 2,82	30,48 ± 1,61
**HW (g)**	0,18 ± 0,03	0,16 ± 0,01
**TL (mm)**	17,26 ± 0,55	17,28 ± 0,44
**IVSd (mm)**	0,67 ± 0,14	0,76 ± 0,13
**IVSs (mm)**	0,71 ± 0,14 $$$$ p<0.0001	0,94 ± 0,25 # p=0.0163
**LVIDd (mm)**	5,46 ± 0,83 $$$ p=0.0002	4,75 ± 0,65 # p=0.0389
**LVIDs (mm)**	5,01 ± 0,91 $$$$ p<0.0001	3,86 ± 0,97 ## p=0.0093
**LVPWd (mm)**	0,81 ± 0,16	0,75 ± 0,1
**LVPWs (mm)**	0,95 ± 0,25	1,02 ± 0,15
**EF (%)**	18,36 ± 8,64 $$$$ p<0.0001	39,22 ± 19,11 ## p=0036
**FS (%)**	8,46 ± 4,14 $$$$ p<0.0001	19,89 ± 11,32 ## p=0.0051
**Heart rate**	404 ± 30	376 ± 60

sham, sham-operated control group; MI, myocardial infarction; BW, body weight; HW, heart weight; TL, tibia length; IVSd, interventricular septal thickness at end-diastole; IVSs, interventricular septal thickness at end-systole; LVIDd, left ventricular internal dimension at end-diastole; LVIDs, left ventricular internal dimension at end-systole; LVPWd, left ventricular posterior wall thickness at end-diastole; LVPWs, left ventricular posterior wall thickness at end-systole; EF, ejection fraction; FS, fractional shortening; Data are expressed as means ± STDEV. Comparison of two groups was performed with the unpaired, two-tailed Student's t-test between Zeb2 WT post-MI vs Zeb2 WT post-sham (indicated by dollar sign, $$$p<0.001, $$$$p<0.0001) and Zeb2 cTg post-MI vs Zeb2 WT post-MI (indicated by pound sign, #p<0.05, ##p<0.01).

Supplementary Table 5. Morphometric and echocardiographic characteristic of sham or MI subjected mice treated with AAV9-control and AAV9-Zeb2 for 14 days.

	AAV9-control	AAV9-Zeb2
**Surgery**	sham	sham
**n**	6	6
**BW (g)**	26,66 ± 2,77	25,75 ± 1,29
**HW (g)**	0,12 ± 0,01	0,13 ± 0,01
**TL (mm)**	16,80 ± 0,37	16,81 ± 0,19
**IVSd (mm)**	0,78 ± 0,09	0,75 ± 0,08
**IVSs (mm)**	1,09 ± 0,11	1,09 ± 0,11
**LVIDd (mm)**	3,86 ± 0,19	3,76 ± 0,19
**LVIDs (mm)**	2,93 ± 0,38	2,85 ± 0,28
**LVPWd (mm)**	0,79 ± 0,08	0,83 ± 0,08
**LVPWs (mm)**	0,95 ± 0,09	1,02 ± 0,12
**EF (%)**	48,56 ±10,69	48,79 ± 9,32
**FS (%)**	24,36 ± 6,42	24,36 ± 5,44
**Heart rate**	398 ± 30	416 ± 20
	AAV9-control	AAV9-Zeb2
**Surgery**	MI	MI
**n**	10	10
**BW (g)**	27,96 ± 1,44	25,87 ± 2,41
**HW (g)**	0,16 ± 0,02	0,16 ± 0,03
**TL (mm)**	16,93 ± 0,44	16,76 ± 0,30
**IVSd (mm)**	0,64 ± 0,07 $$ p=0.0041	0,83 ± 0,13 ### p=0.0009
**IVSs (mm)**	0,68 ± 0,11 $$$$ p<0.0001	0,99 ± 0,19 ### p=0.0003
**LVIDd (mm)**	5,26 ± 0,78 $$$ p=0.0007	4,51 ± 0,58 # p=0.0271
**LVIDs (mm)**	4,69 ± 0,88 $$$ p=0.0003	3,67 ± 0,52 ## p=0.0066
**LVPWd (mm)**	0,75 ± 0,12	0,86 ± 0,08 # p=0.0302
**LVPWs (mm)**	0,89 ± 0,15	1,08 ± 0,11## p=0.0061
**EF (%)**	23,97 ± 9,76 $$$ p=0.0002	38,95 ± 8,43 ## p=0.0019
**FS (%)**	11,21 ± 4,69 $$$ p=0.0002	18,93 ± 4,68 ## p=0.0018
**Heart rate**	403 ± 34	401 ± 66

sham, sham-operated control group; MI, myocardial infarction; BW, body weight; HW, heart weight; TL, tibia lenght; IVSd, interventricular septal thickness at end-diastole; IVSs, interventricular septal thickness at end-systole; LVIDd, left ventricular internal dimension at end-diastole; LVIDs, left ventricular internal dimension at end-systole; LVPWd, left ventricular posterior wall thickness at end-diastole; LVPWs, left ventricular posterior wall thickness at end-systole; EF, ejection fraction; FS, fractional shortening; Data are expressed as means ± STDEV. Comparison of two groups was performed with the unpaired, two-tailed Student's t-test between AAV9-control post-MI vs AAV9-control post-sham (indicated by dollar sign, $$p<0.01, $$$p<0.001, $$$$p<0.0001) and AAV9-Zeb2 post-MI vs AAV9-control post-MI (indicated by pound sign, #p<0.05, ##p<0.01, ###p<0.001).

Supplementary Table 6. Morphometric and echocardiographic characteristic of sham or MI subjected mice treated with AAV9-control and AAV9-Zeb2 for 28 days.

	AAV9-control	AAV9-Zeb2
**Surgery**	sham	sham
**n**	6	6
**BW (g)**	25,61 ± 1,83	28,06 ± 2,12
**HW (g)**	0,14 ± 0,02	0,13 ± 0,01
**TL (mm)**	16,70 ± 0,34	17,04 ± 0,44
**IVSd (mm)**	0,77 ± 0,09	0,77 ± 0,09
**IVSs (mm)**	1,03 ± 0,16	1,05 ± 0,13
**LVIDd (mm)**	3,91 ± 0,55	4,00 ± 0,27
**LVIDs (mm)**	3,20 ± 0,45	3,31 ± 0,33
**LVPWd (mm)**	0,75 ± 0,12	0,79 ± 0,17
**LVPWs (mm)**	0,87 ± 0,10	0,97 ± 0,22
**EF (%)**	38,09 ± 1,57	36,22 ± 10,81
**FS (%)**	18,07 ± 0,94	17,34 ± 6,04
**Heart rate**	426 ± 29	432 ± 54
	AAV9-control	AAV9-Zeb2
**Surgery**	MI	MI
**n**	10	10
**BW (g)**	27,43 ± 2,45	28,95 ± 2,45
**HW (g)**	0,16 ± 0,03	0,17 ± 0,03
**TL (mm)**	17,00 ± 0,28	17,29 ± 0,50
**IVSd (mm)**	0,71 ± 0,13	0,83 ± 0,13 # p=0.0301
**IVSs (mm)**	0,79 ± 0,22 $ p=0.0310	0,96 ± 0,25
**LVIDd (mm)**	5,29 ± 0,89 $$ p=0.0034	5,28 ± 0,78
**LVIDs (mm)**	4,89 ± 0,99 $$ p=0.0012	4,64 ± 0,96
**LVPWd (mm)**	0,82 ± 0,09	0,84 ± 0,12
**LVPWs (mm)**	0,93 ± 0,12	1,04 ± 0,14 # p=0.0413
**EF (%)**	17,27 ± 9,05 $$$$ p<0.0001	26,56 ± 12,24 # p=0.0499
**FS (%)**	7,912 ± 4,28 $$$$ p<0.0001	12,61 # 6,21 # p=0.0451
**Heart rate**	450 ± 59	431 ± 45

sham, sham-operated control group; MI, myocardial infarction; BW, body weight; HW, heart weight; TL, tibia lenght; IVSd, interventricular septal thickness at end-diastole; IVSs, interventricular septal thickness at end-systole; LVIDd, left ventricular internal dimension at end-diastole; LVIDs, left ventricular internal dimension at end-systole; LVPWd, left ventricular posterior wall thickness at end-diastole; LVPWs, left ventricular posterior wall thickness at end-systole; EF, ejection fraction; FS, fractional shortening; Data are expressed as means ± STDEV. Comparison of two groups was performed with the unpaired, two-tailed Student's t-test between AAV9-control post-MI vs AAV9-control post-sham (indicated by dollar sign, $p=0.05, $$p<0.01, $$$$p<0.0001) and AAV9-Zeb2 post-MI vs AAV9-control post-MI (indicated by pound sign, #p<0.05).

Supplementary Table 7. List of primers used in this study.

Gene	Species	Purpose	Forward	Reverse
Zeb2	mouse	QPCR	gagcaggtaaccgcaagttc	aagcgtttcttgcagtttgg
App	mouse	QPCR	gaatggaaagtgggagtcagac	accagttctggatggtcactg
Basp1	mouse	QPCR	cgggagagagagagagcctttg	cttcttcttgctcagcttgcc
Clic1	mouse	QPCR	cacagacaccaacaagatcgag	tttgcaaatatgtccagtcccg
Dynll1	mouse	QPCR	gaaggatattgcggcccatatc	agtgtttggtttcatgtgtcac
Ptma	mouse	QPCR	caccaaggacttgaaggagaag	ctacctcattgtcagcctcctg
Rack1	mouse	QPCR	gaggtcactcccacttcgttag	gacaaatcgccttgtggtg
Spcs2	mouse	QPCR	agaactctctggatgactctgc	atcccaaatcaaagccacgatg
Tapbp	mouse	QPCR	gccagatcttgacccaaagc	gatgaagcggctcatctcg
Tmsb4	mouse	QPCR	aacgcaagagaaaaatcctctg	aaataagaaggcaatgctcgtg
Tmsb10	mouse	QPCR	taaggccaagctgaagaaaacc	gggtcttggagatgatgggg
Ywhab	mouse	QPCR	ggacacgaactctccaatgaag	ctgctgcttcttctcattcctc
Pecam1	mouse	QPCR	gaacggaaggctcccttaat	ggggacaggctcataaatacg
Vegf (all isoforms)	mouse	QPCR	tgcggatcaaacctcaccaaa	ctggctttgttctgtctttctttgg
Sfrp2	mouse	QPCR	gaagcctgcaaaaccaagaatg	cttgctctttgtctccaggatg
Gpnmb	mouse	QPCR	gaatgggatgaacacctgtatcc	ccacaaaagtgatattggaaccc
Thbs1	mouse	QPCR	caaggcattgatctctctctcc	gttgcaaagggagatgtcaatg
Eln	mouse	QPCR	gttcccggtggagtctattatc	ctccaaacgttcccagaagtc
Egf	mouse	QPCR	aagagttttccttaacgggacag	tgactccgttctgttggtctac
Epn3	mouse	QPCR	agtcgcgaagaggctgaaag	cactggagagtcatctcccttc
Myot	mouse	QPCR	aaatgcaaagacacccttcttc	tatgctgtggtgaatcttggac
Mybpc2	mouse	QPCR	atcaagtggttcaaggggaag	acggtgtacacattgctggtag
Gata2	mouse	QPCR	tgtcttcttcaaccatctcgac	gctgtgcaacaagtgtggtc
Acta2	mouse	QPCR	acccacccagagtggagaag	agcatcatcaccagcgaag
Notch1	mouse	QPCR	cccttgctctgcctaacg	gcagacacaggcttcagtgc
Zeb2	rat	QPCR	ccgttggacctgtcattacc	ggatgaagaaacactgttgtgg
App	rat	QPCR	aatggaaaatgggagtcagacc	accagttctggatggtcactg
Basp1	rat	QPCR	gaaggagagcgaaccccag	cttgtcggcctcctttttcctc
Clic1	rat	QPCR	gagtcaccttcaacgttaccac	tgttggtatctgtgtgcacttc
Dynll1	rat	QPCR	cacggtaaccatgtgcgac	tacttctccaacgcctgagtag
Ptma	rat	QPCR	ggaggcagagaatggaagagac	cttcctcaccatcaccttcttc
Rack1	rat	QPCR	tgttagcgatgttgtcatctcc	gacaaatcgtctcgtggtagtg
Spcs2	rat	QPCR	ccgcagtggtttgttggataag	gagaccgaagttctccacatac
Tapbp	rat	QPCR	cggccagatcttgaccctaag	gaacgggatgtagctgctcatc
Tmsb4	rat	QPCR	gatgcaaagaggttggatcaag	catgcaagttcttttccctctc
Tmsb10	rat	QPCR	taaggccaagctgaagaaaacc	ttcttggagatgaaggggtgg
Ywhab	rat	QPCR	gtaacagagcagggacatgaac	ctgctgcttcttctcattcctc
Col1a2	rat	QPCR	tggaaaacctggaagacctg	atccagaccgttgtgtcctc
Col3a1	rat	QPCR	agctggaccaaaaggtgatg	gacctcgtgctccagttagc
Bcl2	rat	QPCR	gtggacaacatcgctctgtg	ctgagcagcgtcttcagaga
Vegf (all isoforms)	rat	QPCR	gcggatcaaacctcaccaaag	tggctttgttctatctttctttgg
Pecam1	rat	QPCR	atatcagcaccacctcgaaatc	agactgaggaatgacgaagctc
ZEB2	human	QPCR	gacattccagaaaagcagttcc	gaagccttgagtgctcgataag
TMSB4	human	QPCR	aacgcaagagaaaaatcctctg	aaataagaaggcaatgctcgtg
PTMA	human	QPCR	caccaccaaggacttaaaggag	cttcgtctacctcattgtcagc
VEGFR1	human	QPCR	gctcagctgtctgcttctcac	agaccatttatgggctgcttc
VEGFR2	human	QPCR	caggatgcagagcaaggtg	ctgggcagatcaagagaaacac
FGF2	human	QPCR	tgccaaggctgaaattatcc	ctccgttcctcatccaaaag
NOTCH1	human	QPCR	ggaggcatcctacccttttc	tgtgttgctggagcatcttc
MIKI67	human	QPCR	aaaaggattccctcagcaagcc	ccctcactctcattaatggaatcac
PCNA	human	QPCR	gaacctcaccagtatgtccaaaatac	ctttctcctggtttggtgcttc
CDK4	human	QPCR	aattgcatcgttcaccgagatctg	gagtgtaacaaccacgggtgtaa
Cre		genotyping	gaagcaactcatcgattgatttacg	cactatccaggttacggatatagttc
Zeb2 floxed allele		genotyping	(intron 6) gaactagttgaattggtagaatcaatgggg	(intron 6) atcagcagcctcctatttaaacagagtgtc
		genotyping		(intron 7) aagcatgtcggtaagctgaccaactactag
Zeb2 knock in		genotyping	aaagtcgctctgagttgttat	(wild type) ggagcgggagaaatggatatg
		genotyping		(knock in) gcgaagagtttgtcctcaacc
