# Supplementary material for: Cardiomyocytes stimulate angiogenesis after ischemic injury in a ZEB2-dependent manner
Source: Nat Commun. 2021 Jan 4;12:84. doi: 10.1038/s41467-020-20361-3 (PMC7782784; doi:10.1038/s41467-020-20361-3)
Supplement: Supplementary file 3 — Description of Additional Supplementary Files [file 41467_2020_20361_MOESM3_ESM.docx]

**Supplementary information**

**Cardiomyocytes stimulate angiogenesis after ischemic injury in a ZEB2-dependent manner**

**Authors:** Monika M. Gladka^1^, Arwa Kohela^1^, Bas Molenaar^1^, Danielle Versteeg^1,2^, Lieneke Kooijman^1^, Jantine Monshouwer-Kloots^1^, Veerle Kremer^3,4^, Harmjan R. Vos^5^, Manon M.H. Huibers^6^, Jody J. Haigh^7^, Danny Huylebroeck^8,9^, Reinier A. Boon^3,10,11^ Mauro Giacca^12^, Eva van Rooij^1,2^*

**Affiliations:**

^1^ Hubrecht Institute, Royal Netherlands Academy of Arts and Sciences (KNAW) and University Medical Centre, Utrecht, The Netherlands

^2^ Department of Cardiology, University Medical Center, Utrecht, The Netherlands

^3^ Department of Physiology, Amsterdam University Medical Center VU, The Netherlands

^4^ Department of Medical Biochemistry, Amsterdam University Medical Center, The Netherlands

^5^ Molecular Cancer Research, Center for Molecular Medicine, University Medical Center, Utrecht, The Netherlands

^6^ Department of Pathology, University Medical Centre Utrecht, The Netherlands

^7^ Department of Pharmacology and Therapeutics, University of Manitoba, Canada

^8^ Department of Cell Biology, Erasmus University Medical Centre, Rotterdam, The Netherlands

^9^ Department of Development and Regeneration, University of Leuven, Belgium

^10^ Institute for Cardiovascular Regeneration, Centre for Molecular Medicine, Goethe University, Frankfurt am Main, Germany

^11^ German Center for Cardiovascular Research (DZHK), Frankfurt am Main, Germany

^12^ School of Cardiovascular Medicine and Sciences, King’s College London, UK

**Content**

- Supplementary Data Legends

**Supplementary Data Legends**

**Supplementary Data 1.** Gene profiling of all clusters identified by single-cell sequencing. The first sheet shows an overview of all the identified clusters and the number of cells per cluster. Following sheets show the gene expression signature of all 14 clusters. For quantification of transcripts abundance, the number of transcripts containing unique molecular identifiers per cell-specific barcode was counted for each gene. K-medoids clustering was based on the RaceID2 algorithm to visualize cell clusters using t-distributed stochastic neighbor embedding (t-SNE) and to compute genes up- or downregulated in all cells within the cluster compared with cells not in the cluster.^1,2^ . Data sorted based on fold change.

**Supplementary Data 2.** List of genes enriched in cardiomyocytes from injured heart compared to sham cardiomyocytes obtained from the single-cell sequencing analysis. RaceID2 was used to compute genes up- or downregulated in injured cardiomyocytes within compared with healthy carddiomyocytes.^1,2^ Data sorted based on log2 fold change.

**Supplementary Data 3.** RNA-seq-based gene profiling of *Zeb2* fl/fl and *Zeb2* cKO hearts 14 dpMI. Differential expression was analysed by DESeq v1.22^3^ using per condition dispersion estimates.

**Supplementary Data 4.** List of proteins detected by mass-spec as shown in details in Figure 3. The first sheet shows all detected proteins. The second sheet shows the top 40 proteins that are classified as plasma proteins based on Human Protein Atlas. The third sheet shows 11 proteins that were also downregulated in RNA-seq form Zeb2 cKO mice vs Zeb2 fl/fl post-MI, as shown in details in Supplementary Figure 4.

References:

1 Grun, D. *et al.* De Novo Prediction of Stem Cell Identity using Single-Cell Transcriptome Data. *Cell Stem Cell* **19**, 266-277, doi:10.1016/j.stem.2016.05.010 (2016).

2 Grun, D. & van Oudenaarden, A. Design and Analysis of Single-Cell Sequencing Experiments. *Cell* **163**, 799-810, doi:10.1016/j.cell.2015.10.039 (2015).

3 Anders, S. & Huber, W. Differential expression analysis for sequence count data. *Genome Biol* **11**, R106, doi:10.1186/gb-2010-11-10-r106 (2010).
